# Intensification of camel farming and milk production with special emphasis on animal health, welfare, and the biotechnology of reproduction

**DOI:** 10.1093/af/vfac043

**Published:** 2022-08-12

**Authors:** Peter Pal Nagy, Julian Alexandra Skidmore, Judit Juhasz

**Affiliations:** Farm and Veterinary Department, Emirates Industry for Camel Milk and Products, Dubai, United Arab Emirates; Camel Reproduction Centre, Dubai, United Arab Emirates; Farm and Veterinary Department, Emirates Industry for Camel Milk and Products, Dubai, United Arab Emirates

**Keywords:** animal health, dromedary camel, intensification, milking, reproduction, welfare

**Implications**

- Dromedaries can be integrated efficiently into an intensive production environment.
- Intensive camel milk production requires a significant initial investment, however, it also offers a number of advantages.
- A Herd Health Management program and compliance with statutory requirements guaranty the production of good quality and safe raw camel milk from “*happy and healthy*” animals.
- No undesired effects associated with intensification such as the emergence of zoonotic diseases and antibiotic resistance were detected.
- Assisted reproductive technologies are important to enhance the efficiency of a selective breeding program.

## Sustainable Development, Intensification, and the Role of Camels in Food Security in Arid and Semiarid Regions

The growing population of the world is projected to reach 9.7 billion by the year 2050. Food and water security are among the priorities of the 21st century that cannot be achieved without sustainable and intensive agricultural production. Intensification of both crop and livestock production started some decades ago and resulted in the so-called “green revolution” in crop production and in the expansion of livestock production – the “livestock revolution” (Gilbert et al., 2021). Recently, the international community set out 17 “sustainable development goals” (**SDGs**) to ensure development of the world as a whole in the coming years. Among those, SDG-2 aims to “end hunger, achieve food security and improved nutrition and promote sustainable agriculture” through determining targets and indicators (Gil et al., 2019). One of these targets is to double agricultural productivity of small-scale farms. In parallel with intensification, we also have to consider the effect of climate change on the environment and on agricultural production. We can expect profound changes in the sustainability of various livestock systems mainly in the grazing/pastoral and in the mixed crop-livestock production systems due to decreased forage quantity and quality, increased heat stress, and animal health problems (Nardone et al., 2010). Although, the intensification of livestock production is necessary and essential to reach SDG targets, it also has some risks and unwanted effects that should be mitigated. These are the emergence of zoonotic diseases, the concentration of livestock in peri-urban areas, environmental degradation, and the spread of antimicrobial resistance (Gilbert et al., 2021).

The number of camels has been continuously increasing in the last few decades and has reached approximately 40 million head of Bactrian and dromedary camels and this number is expected to increase to above 60 million in 25-years’ time (Faye, 2020). In parallel with the number of animals, the world’s annual camel milk production has also increased from 0.63 million tonnes in 1961 to 3.15 million tonnes in 2020 (FAO, 2020; https://www.fao.org/faostat/en/#data/QCL); this is a 5-fold increase over the 60-yr period. With this quantity, camels are the fifth most important dairy animals, following cattle, water buffalo, goat, and sheep (Faye and Konuspayeva, 2012). However, until recently, camel milk had been produced exclusively by hand milking in traditional, extensive nomadic, or semiintensive farming systems, and such production could not maintain constant quantity and quality of raw milk for urban markets (Abeiderrahmane, 2005). In addition, most of the production had been consumed locally, without any quality control or further processing, and, therefore, camel milk has not yet been widely integrated into national and international markets (Faye et al., 2014). Moreover, camels were regarded mainly as pack or racing animals by many people including the general public, scientists, funding agencies, and policy makers. As a result, hardly any efforts have been made to intensify milk production and improve production traits in dromedaries.

This situation is likely to change and the intensification of camel farming is expected to take place in the coming decades for several reasons. In addition to the growing world population and increased demand for products of animal origin in general, the public awareness of camel milk and meat is also increasing. At the same time agricultural practices will be affected by decreasing water resources and land desertification as a result of climate change allowing livestock species well adapted to arid environments to gain more space (Nardone et al., 2010; Kagunyu and Wanjohi, 2014; Gilbert el al., 2021). The first steps of intensification of the camel dairy industry started 15 to 20 yr ago (Nagy and Juhász, 2016). Machine milking has been introduced in several traditional camel keeping countries like Tunisia, Saudi Arabia, and the United Arab Emirates (Wernery et al., 2004; Hammadi et al., 2010; Ayadi et al., 2013). Small-scale farms in Australia, Europe, and the United States have also started using milking machines for dromedaries. The world’s first large-scale camel dairy farm (Emirates Industry for Camel Milk and Products, **EICMP**) with processing and distribution facilities has also been established during this period (Juhasz and Nagy, 2012a; Nagy et al., 2013b). The aim of the project was to develop a biologically, environmentally, and financially sustainable, intensive camel milk production system and to meet the quantity and quality requirements of the market. It is evident that these goals could not be achieved without systematic research and development, including all aspects of camel behavior, nutrition, lactation physiology, reproduction, husbandry, and management, taking into account the special characteristics and requirements of this unique species.

The aim of this paper is to review our experience on the present status and challenges related to the intensification of camel farming. The large pool of animals at EICMP and Camel Reproduction Centers (**CRC**) together with the solid data generated by the operations have provided an unprecedented opportunity for developing, monitoring, and improving an intensive camel milk production system for dromedaries. Here, we focus mainly on animal health, welfare, and management as well as on assisted reproduction.

## Milk Production Potential of Dromedaries

Earlier, the production potential of dromedaries was evaluated by hand-milking, with results showing great variability that were difficult to compare. This was partly due to the fact that authors used various measurement procedures and estimation for milk production, such as the calf suckling method, hand milking of two or four quarters, and the milk oxytocin technique (Simpkin and Rowlinson, 1998; Jemmali et al., 2016). Frequently, the estimated quantity of milk consumed by the calf was also added to the daily yield. Moreover, milk production has been expressed in different units such as daily or weekly average, daily maximum, total lactation or 305-day production, or herd average. Therefore, individual total production was reported ranging from 1,000 to 12,000 liters during an 8 to 18 months’ lactation period with significant variations between geographical regions (Africa vs. Asia) and daily maximum production reached as high as 35 to 40 liters (Faye, 2008). Recently, several detailed studies were conducted on the milk yield of dromedaries in various countries and a standardized milk recording method was proposed (Boujenane, 2020). Musaad et al. (2013) reported a lactation average of 2,220 ± 925 liters during a 12.5 mo lactation period in mixed breed dromedaries (*n* = 47) in Saudi Arabia. In contrast, the production potential of Maghrebi camels (*n* = 10) in Egypt was lower, with an average production of 1612 ± 710 liters during 353 ± 152 d lactation period (Abdalla et al., 2015). The total milk yield of Tunisian camels in one study (*n* = 8) was an average 2,642 ± 523 liters for 390 d of lactation (Jemmali et al., 2016), while in another study (*n* = 95) it was 1,388 ± 575 liters for 11 mo of lactation (Chamekh et al., 2020). At EICMP in the UAE, the mean total production was 3,152 ± 73.5 kg for an average lactation period of 585 ± 11.0 d in 385 machine-milked dromedaries and the daily maximum rarely exceeded 25 kg. The lactation curve showed a high persistency (>93%), with peak production during the 4th month after parturition, after which mean yield declined gradually reaching 50% of the maximum by the 16th month postpartum (Nagy and Juhasz, 2016). Total milk yield is influenced by several factors such as breed, lactation length, parity, calving season, year, milking frequency, milking method, photoperiod, production system, and pregnancy (Nagy and Juhász, 2016, Boujenane, 2020).

## Advantages and Disadvantages of Intensification of Camel Milk Production

Although most camels are kept in developing countries under pastoral, extensive, or semiintensive systems, well-planned intensification might potentially help the further development of the species and its integration into the food production chain (Faye et al., 2014; Faye, 2020). Some may look at intensive farming as an unnatural and harmful environment for dromedaries that restricts the free movement of the animals and the expression of typical behavior. Such people tend to focus mainly on its real or assumed negative aspects. Indeed, intensive camel milk production requires a significant initial investment to build the proper infrastructure for a sufficient number of animals with enough paddock space and a regular and reliable feed supply and continuous water source as well as well-trained professionals and veterinary service to tend the animals. It would also need to include a milking facility unit with cooling system and a sufficient milk storage area both having a continuous electricity supply. Also, producers must have reliable access to markets to be able to sell the milk. Unfortunately, today these requirements are difficult to meet in most places where camels are kept naturally. In addition, intensive production has a substantial environmental impact compared to pasture-based systems, therefore manure management must also be taken into consideration. The concentration of animals and husbandry practices could lead to the increased emergence of noninfectious and infectious diseases and antimicrobial resistance (Gilbert el al., 2021). On the other hand, if the above conditions are available, intensive production can offer a number of advantages. Primarily, it allows the efficient and cost-effective production of high quality, raw camel milk that is suitable for further processing and meets the quality requirements of the consumers of the 21st century. At the same time, such a production also ensures that the animal health and animal welfare requirements of the species are met. This is achieved by adhering to national and international guidelines, statutory requirements, and standards.

One of the main advantages of modern and intensified animal production is that it is well regulated. In response to the 1965 UK Government report on livestock husbandry, the UK Farm Animal Welfare Council published its recommendations in a press release in 1979 that became known as the Five Freedoms of animal welfare (FAWC, 1979). These recommendations have been adopted by professional groups and international organizations (i.e., FAO, OAI) and integrated into their standards. Today, food safety control measures at farm level are typically described by best practice techniques such as good farming practices (**GFP**) and good veterinary practices (**GVP**). The role and responsibilities of international organizations such as the Codex Alimentarius Commission (**CAC**), World Organization of Animal Health (**OAI**), and Food and Agriculture Organization (**FAO**) are to set standards and give recommendations on various aspects of agricultural production (FAO – farming practices; OAI – animal health; CAC – food safety). These standards have been integrated by the International Organization for Standardization (**ISO**) into a comprehensive food safety management system (**FSMS**) that can be applied voluntarily at farm level (ISO22000: 2018). However, before such a FSMS is implemented, so-called prerequisite programs (**PRPs**) need to be established. The currently existing PRPs on food safety of farming are detailed in a separate standard (ISO/TS 22002-3:2011). This comprehensive document details all the general requirements of farming including the location, construction and layout of premises, equipment suitability and maintenance, personal hygiene, management of procurements, on-farm storage and transport, cleaning, waste management, pest control, and the use of unsafe products. In addition, it also contains specific requirements for animal production such as feeding and watering, identification and movement of animals, health monitoring, handling of sick and dead animals, the use of veterinary drugs, milking, and slaughter. Most of these requirements would be difficult to meet in a pastoral, extensive production system. However, any milk producing establishment complying with local/national statutory requirements and the above standards is able to produce safe milk for human consumption and, at the same time, fulfil the needs of the animals. EICMP has been successfully running an ISO FSMS since 2009.

## Physiological and Behavioral Characteristics of Dromedaries That Should be Taken Into Account for Intensive Production and the Development of the Infrastructure

It is widely documented that camels are well adapted to arid to semiarid conditions and harsh environment. Their upper limit of the so-called thermoneutral zone is approximately 40 °C and healthy animals are not sensitive to “heat stress” under normal watering conditions. Camels minimize water losses with various physiological mechanisms such as adaptive hyperthermia in dehydrated camels, brain cooling, nasal heat exchange, decreased and concentrated urine excretion, way of urination, and dry feces; they have high tolerance to dehydration and can rehydrate rapidly after prolonged water deprivation (Wilson, 1989; Hoter et al., 2019). However, we have to emphasize that in properly watered animals – that is a prerequisite in a camel dairy – body temperature changes within narrow limits between approximately 36 °C and 38 °C. Therefore, elevated body temperature above 38.5 °C is not a sign of adaptive hyperthermia and not physiological, rather it is a sign of disease and requires veterinary attention. The daily water requirement of camels is very low, it was calculated by Schmidt-Nielsen et al. (1956) to be 4.9 liters/100 kg body weight (bw). Based on this calculation we estimated the daily water requirement of a 620 kg lactating dromedary giving 7.2 kg of milk/day to be approximately 37 liters, while the actual consumption was 45.6 liters of water a day (P. Nagy and J. Juhasz, personal communication). In addition, camels have an efficient feed conversion, therefore the requirement of dry matter intake for maintenance is approximately 1% of bw, but a 620 kg lactating dromedary requires approximately 2% of bw dry matter intake daily for the above-mentioned daily milk production. However, under natural conditions camels spend a significant amount of time grazing, browsing, and searching for feed in the desert which is not the case under intensive farming conditions (Iqbal and Khan, 2001).

The natural and typical behavior of the species should be taken into account when developing and managing an intensive production system. Camels are social, calm, and peaceful herd animals that have a strong bond within the group and also with their offspring. The physical and visual contact with the calf is vital to maintain milk production, therefore, weaning the calf (complete physical separation) occurs at a much later stage (7 to 12 mo). Frequently, camels have a reputation as being aggressive and bad-tempered, and as being difficult and dangerous to handle. However, if trained and handled properly camels can become companion animals attached to their caretakers and they can equally adapt to a nonpersonal, large-scale husbandry system. This is due to the supposed aptitude, high cognitive function, sophisticated mental capacity, and learning ability of the species. This statement is based on our personal experience and is not supported by actual research data in this species. However, cognitive capacity in other livestock species has been already demonstrated and is a new area of research for animal welfare and for improving livestock management (Nawroth et al., 2019).

**Figure 1.**
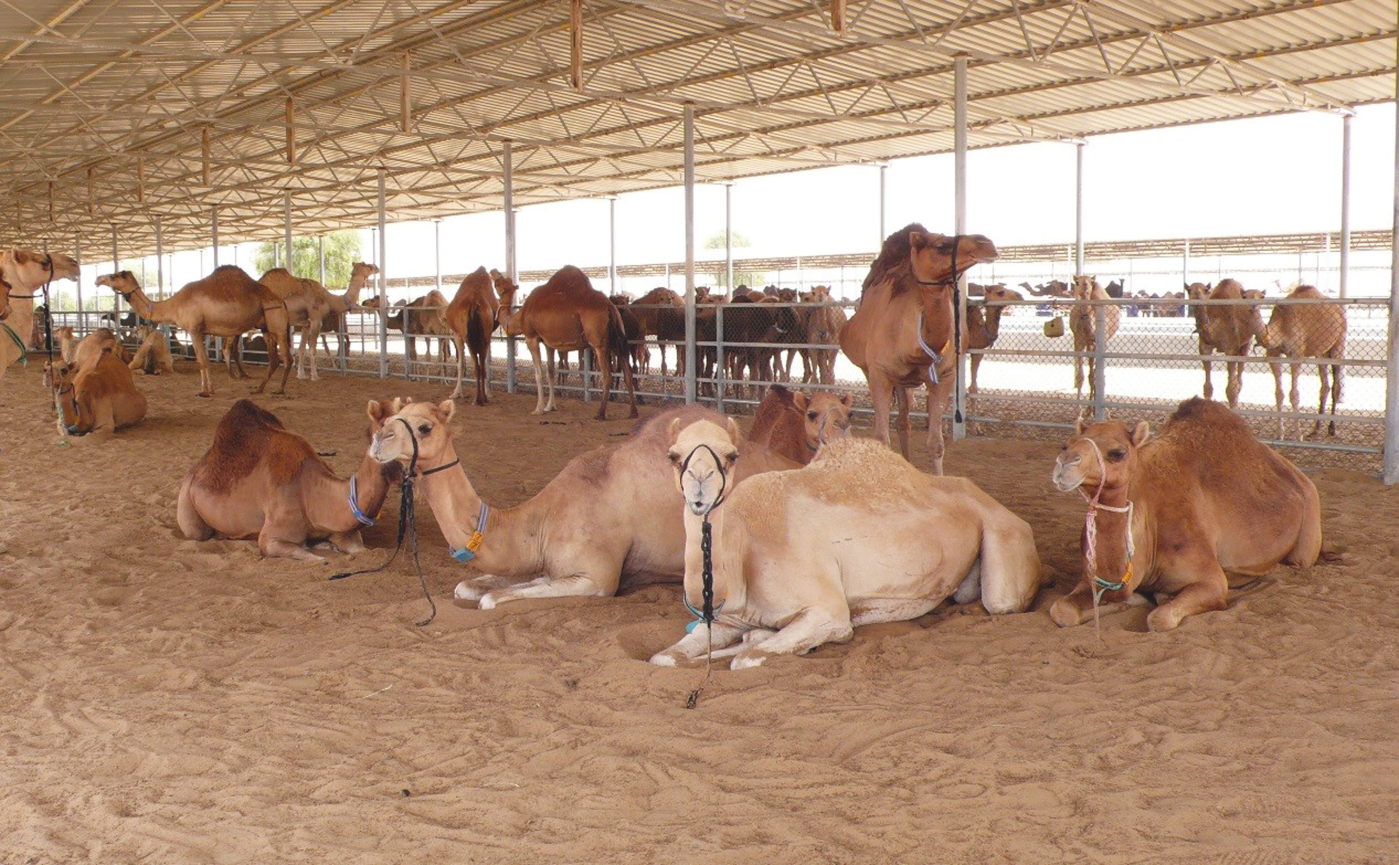
Lactating dromedaries in spacious paddock under shade and their calves in adjacent paddock.

**Figure 2.**
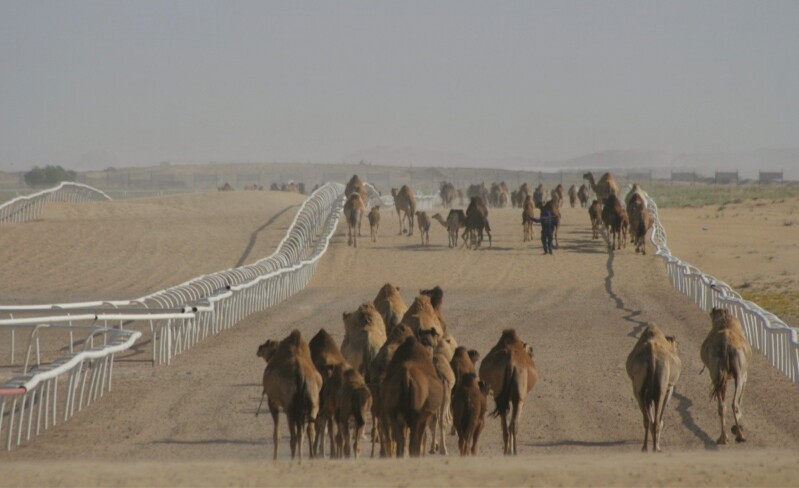
Dromedaries and their calves on the walking track during their daily exercise.

All the above parameters should be considered when developing the premises of an intensive farm. The infrastructure of camel husbandry can be rather simple under the climatic conditions of the UAE and most camel keeping countries. Although dromedaries should be kept in spacious paddocks with shades, they do not require any cooling technology even during the summer period as opposed to dairy cows in the same environment. We calculate approximately 50 m^2^ paddock space for a lactating dromedary of which approximately 30% (15 m^2^) is shaded (Figure 1). These dimensions provide sufficient individual space within the herd and larger paddocks, especially as larger shaded areas would not be utilized efficiently by the group. Water is available constantly, but temperature control is not necessary. However, it is important to provide sufficient feeding space for all animals in the group to feed at the same time. Therefore, we calculate approximately 80 to 100 cm feeding space per animal to avoid competition and fighting. The paddocks of the calves are located adjacent to that of their dams to allow contact, but prevent free suckling of the calves (Figure 1). For mass treatment and handling of the animals, treatment areas (or so-called catching areas) are installed in most paddocks that allow herding, selecting, separating, and individually treating the camels. Compared to pastoral systems where camels are allowed to roam freely, intensive production significantly restricts the movement of animals. In order to mitigate the suspected negative effect of this confinement, at EICMP we have developed exercise facilities, namely walking tracks, where dromedaries have daily controlled exercise for approximately 1 h (Figure 2). A horse walker was also installed for the male dromedaries allowing them to exercise separately.

**Figure 3.**
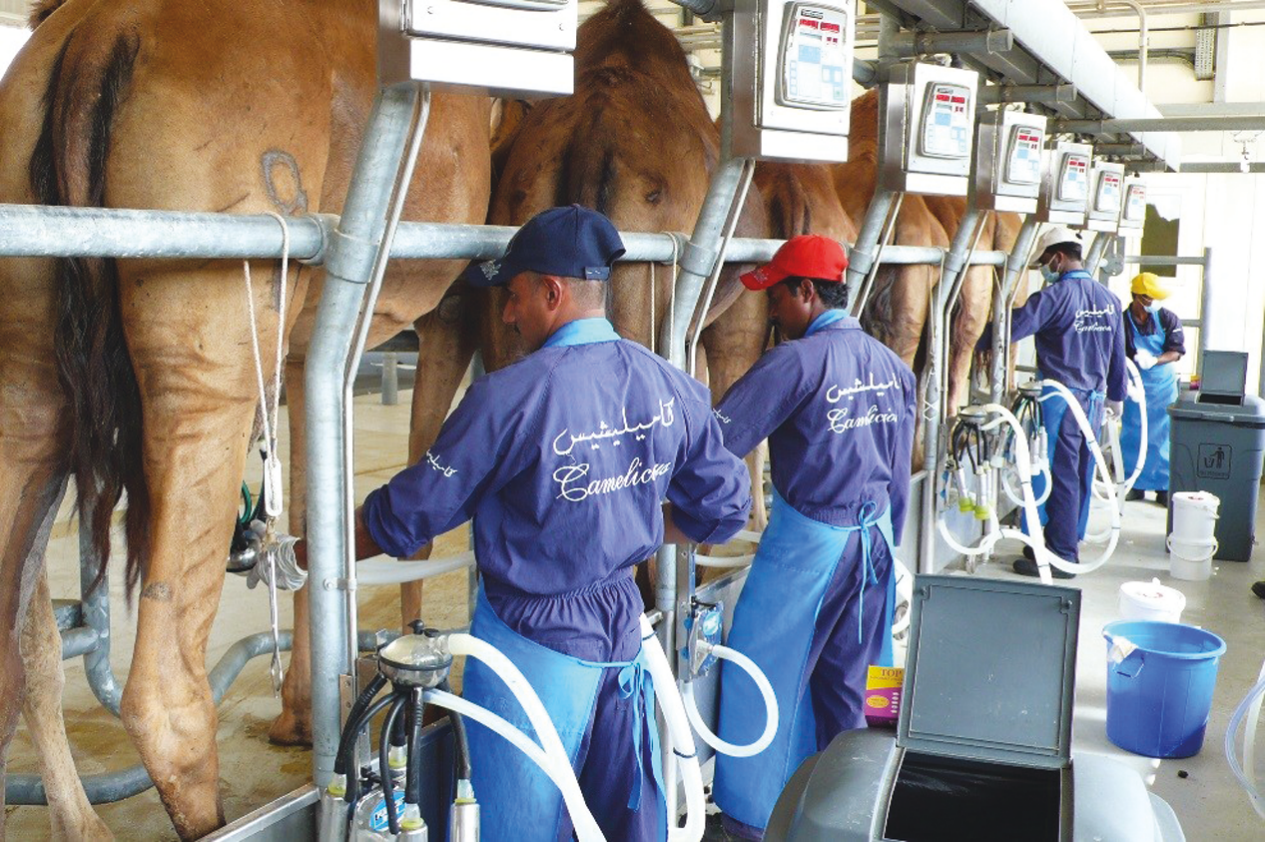
Dromedaries being milked by trained staff in a herringbone milking parlour.

The construction of an efficient and ergonomic milking parlor is crucial for the operation of an intensive camel dairy. Depending on the available resources, various designs can be used ranging from a simple setting to a state-of-the art facility (with identification, milk recording, and milk testing incorporated into a farm management software). However, irrespective of the available technology, the milking stand should be safe and comfortable for both animals and people. Milkers should have easy access to the udder and the flooring of the parlor should be nonslippery to avoid injury of the animals (Figure 3). It is a typical defensive behavior of camels to sit down if there is no other way to escape from a difficult situation, so the design of the parlor should support the effective handling in such an emergency situation. The walkways should also be planned well in order to allow the fast and safe movement of the various groups to and from the milking parlor.

## Animal and Public Health Aspects of Intensive Camel Milk Production

The underlying concept of our intensive production system is that only “*happy and healthy*” camels are able to produce good quality milk close to the maximum of their genetic potential. In order to reach this aim, a comprehensive so-called Herd Health Management program has been implemented and is in practice at EICMP. This program has three main elements which include an animal health and biosecurity program, an animal welfare or well-being program, and a breeding and reproductive management program. These elements are not independent of each other, rather they are very much interlinked sometimes are overlapping, and could not be managed successfully on their own without the other elements. For example, whilst it is easy to understand that the good, healthy condition of pregnant camels cannot be guaranteed without proper reproductive management and animal welfare activities, it is also the case that any diseases in dromedary herds compromise their welfare.

The Animal Health and Biosecurity programs ensure that most camels remain healthy at any given time, the occurrence of new diseases is prevented and sick animals are recognized quickly, and treated efficiently. In a large-scale system, the emphasis is more on prevention rather than on treatment, but looking after individual animals is still an important part of the veterinary service. The Bio-security program is designed to prevent and control any bio-security hazard from entering the premises and spreading on the farm in order to protect the health status of not only the animals, but also of the people. It consists of controlled movement of animals, including quarantine procedures, separation of sick animals, handling of carcasses, cleaning, disinfection, and sanitation of various places on the farm, pest control (rodents, insects, birds), training and healthcare for staff, and the control of movement of people, including visitors and vehicles, into and out of the premises. The Animal health program focuses on three major areas: on infectious disease control, on milking hygiene and mastitis control, and on general animal health which also includes multifactorial or noninfectious diseases and ecto-parasite control. The OAI Terrestrial Animal Health Code (OAI, 2021) describes several so-called outcome-based or animal-based criteria in various livestock species that are useful indicators of animal welfare (and animal health). Dromedaries are not mentioned specifically, but the criteria for dairy cattle can be applied to this species as well. These measurable criteria are suitable to monitor and evaluate the efficiency of the Herd Health Management program and include parameters such as morbidity rate, mortality and culling rates, body condition and milk yield, physical appearance, reproductive parameters, behavior and handling response, and complications from common procedures. Since the beginning of the operation at EICMP, most of the above-mentioned criteria have been systematically recorded. In addition, in the FSMS so-called “measurable food safety objectives” which focus on animal and public health aspects have been determined. The two, animal health related objectives are 1) to keep the occurrence of new clinical mastitis cases per month below 5% and 2) to reduce the less than 1-yr-old calf mortality rate below 10%. Public health related objectives include bulk milk total viable (**TVC**) and Coliform counts (CC) below 10,000 cf/ml and 10 cf/ml, respectively, and the milk free from antibiotic residues.

It is not possible in this manuscript to go into detail for all animal health topics but two of the most important ones such as the Brucella monitoring and the mastitis control programs will be discussed. It is well known that Brucellosis, mainly caused by *Brucella melitensis* bacteria, is the most important zoonotic infectious disease of dromedaries and in certain countries, the seroprevalence can reach as high as 40% (Wernery, 2016). High seropositivity (30%) was also detected in some newly arrived animals at EICMP. Therefore, in addition to strict quarantine procedures, a stringent Brucella monitoring program for the entire herd has been developed at EICMP that comprises frequent on-site and off-site serological screening with Rose Bengal test (**RBT**) and milk ring test (**MRT**). In case of a positive reaction, confirmatory laboratory tests are carried out in the nearby veterinary laboratory according to OAI guidelines in order to exclude false positive animals. Although, over the years there have been sporadic, individual cases of Brucellosis, only one confirmed abortion was detected and the seroprevalence of the herd remained below 0.1% (Juhász et al., 2019). Epidemiological investigations have also been conducted by comparing the genotypes of the different isolates. Disappointingly, no link between the different strains was found, and the source of infections could not be determined (Gyuranecz et al., 2016). It should be emphasized that with strict quarantine procedures and testing, the infection can be effectively prevented from spreading into the main production herd.

As in other species, mastitis is an important and frequent disease in machine milked dromedaries that require continuous monitoring and control. Therefore, a milking hygiene and mastitis control program has been implemented at EICMP. This program includes regular milking parlor maintenance, continuous staff training on milking routines, pest control, daily testing of bulk milk quality, and the regular sampling of all lactating animals at monthly intervals. Milk samples are processed in the in-house laboratory to determine bulk milk TVC and CC and to diagnose animals infected with mastitis pathogenic bacteria. Animals affected with pathogens without clinical signs of mastitis are considered to have subclinical mastitis (Juhász and Nagy, 2012b). In the camel herd, the most important obligate and facultative pathogens are *Streptococcus agalactiae*, *Alpha hemolytic streptococci*, *Streptococcus bovis*, *Coagulase-negative staphylococci*, *Escherichia coli*, *Klebsiella pneumonia*, and *Streptococcus equi subsp. zooepidemicus* (Juhász et al., 2008). The distribution of these bacteria is, however, constantly changing. In cases of both clinical and subclinical mastitis, dromedaries are treated with intramammary and parenteral antibiotics using well defined treatment protocols, and the efficiency of all treatments is evaluated both clinically and microbiologically. If the treatment is not effective (no clinical and microbiological cure), the antibiotic sensitivity of the isolated bacteria is verified and the treatment is repeated accordingly. The milk is only collected for human consumption if the dromedary is clinically healthy and the milk is free from pathogens and antibiotic residues. As mentioned above, the target with this mastitis control program is to keep the incidence of clinical mastitis cases below 5% of the lactating animals per month. During a 12-year period, from 2009 to 2020 the average monthly rate of clinical mastitis was 2.0% although there was some fluctuation between different years and months. The highest and lowest incidence of clinical mastitis are observed in the spring from March to May (2.7% to 3.0%) and in the autumn from September to November (1.2% to 1.6%), respectively.

The most important measurable criteria to evaluate the health status of a livestock production system are the mortality and morbidity rates (OAI, 2021). At EICMP, the average annual mortality rate is 2.45%, while the average morbidity rate is 25.81%. It is important to emphasize that these figures comprise data not only from the production herd, but also from newly arrived camels in quarantine that have a higher incidence of diseases. The most frequent disease is clinical mastitis (29.7%), followed by abscess formation caused by *Corynebacterium pseudotuberculosis* (13.6%), digestive and alimentary tract problems (12.5%), reproductive disorders including abortions, perinatal mortality, postpartum problems (12.5%), and generalized infections with fever (11.7%). Injuries (8.7%), lameness (3.5%), and respiratory tract conditions (2.2%) occur less frequently and the remaining miscellaneous diseases (5.6%) include eye and ear infections, generalized oedema, tympani, foot cancer, neurological syndromes, camel pox, Trypanosomiasis, and snake bites. We also want to mention that common metabolic conditions in dairy cows such as milk fever, ketosis, and fatty liver disease are not observed in dairy camels.

Most of the problems in calves occur when they are less than a year old. In this age group the overall average mortality rate is 13.7% in the herd at EICMP, however it has decreased below 10% in the last five breeding seasons. The most frequent causes of calf death have been white muscle disease, various forms of *E. coli* infection, Clostridiosis, colon fecal impaction, and gastric ulcer. The distribution of the various diseases both in adults and calves is changing from year to year and these figures are difficult to compare due to lack of information in the literature. In pastoral, extensive systems most of these data are not recorded and the focus is mainly on infectious diseases (Dahiya et al., 2016; Wernery, 2016; Salah et al., 2019). For intensive production, there is only one report available in the literature on diseases and mortality in a camel dairy farm in Saudi Arabia (Agab, 2006).

A major advantage of intensive camel milk production in the Middle-East is the controlled use of veterinary drugs where no medicine can be applied without prescription and supervision by a qualified veterinarian. In addition, the return of previously treated animals to production is also strictly controlled using an in-house monitoring program as the milk must be free from residues. Unfortunately, as most commercial veterinary products are not registered for use in lactating camels, the withdrawal periods provided by the manufacturers cannot be applied. According to the relevant EU legislation (Directive 2001/82/EC) the use of nonregistered medicines is considered “off label” and the minimum withdrawal periods for milk and meat are 7 and 28 d, respectively. However, frequently camel milk still contains antibiotic residues 7 d after the end of the treatment. In one of our studies, the milk of 29% of treated dromedaries contained antibiotic residues 7 d posttreatment and the longest withdrawal period was 44 d after an intramammary and parenteral combination. The withdrawal periods were affected by the route of administration, the disease, and also by the type of medicine (Juhász et al., 2015). Unfortunately, few available studies suggest that the withdrawal period of veterinary drugs in dromedary camels is considerably longer than what is recommended for dairy cattle (Eleman et al., 2010; Jayazeri et al., 2012; Wasfi et al., 2012). Therefore, commercial camel dairy operations must develop their own testing protocols or apply sufficiently long waiting periods in order to avoid antibiotic/drug contamination of raw camel milk. Drug metabolism and pharmacokinetics in camels, particularly in lactating camels, require more attention and research.

One of the unfavorable effects of the intensification in livestock production is the spread of antimicrobial resistance (Gilbert et al., 2021). Such an antibiotic (tetracycline) resistance of *Streptococcus agalactiae* strains isolated from camel mastitis cases was described already in Africa and was attributed to the uncontrolled distribution and usage of antibiotics to treat bacterial infections (Fischer et al., 2013). In the production system at EICMP, a lot of effort is taken to prevent the development, and spread of such antibiotic resistance. First of all, only good quality veterinary products, from reputed manufacturers, are purchased through approved suppliers/traders that can guarantee appropriate storage and transport of medicines. Antibiotics are only used if justified by the condition of the animal and no growth promoters are applied for enhancing production. The first choice is to use a broad-spectrum antibiotic, alone or in combination, and targeted treatments are only carried out after the verification of antibiotic sensitivity of the pathogenic bacteria. The efficiency of treatment is always evaluated and if there is not sufficient improvement, the treatment protocol is changed. In case of clinical and subclinical mastitis, the recovery from infection is evaluated not only clinically, but also microbiologically and, if the pathogenic bacteria are still present in the milk, the treatment is repeated. With these measures, except for a few sporadic cases of neonatal infections caused by multiresistance *E. coli*, the development of antimicrobial resistance at EICMP has been prevented.

## Welfare of Dromedaries in Intensive Camel Milk Production

Since the introduction of the Five Freedoms of animal welfare in 1979 (FAWC, 1979), the well-being of livestock species receives more and more attention. This is partly due to pressure from the general public. However, the professional community also recognized and addressed the importance of welfare in livestock production. An entire section of the OAI Terrestrial Animal Health Code (Section 7; OAI, 2021) is dedicated to this subject and it provides comprehensive recommendations for various animal production systems. The welfare aspects of camel husbandry have not been focused on until recently. Although the management and handling of dromedaries in pastoral communities has been described previously (Dioli et al., 1992), studies that systematically assess welfare in this species were only published last year. However, this data was not collected from intensive farms, but rather from a traditional camel market (Menchetti et al., 2021a, 2021b).

**Figure 4.**
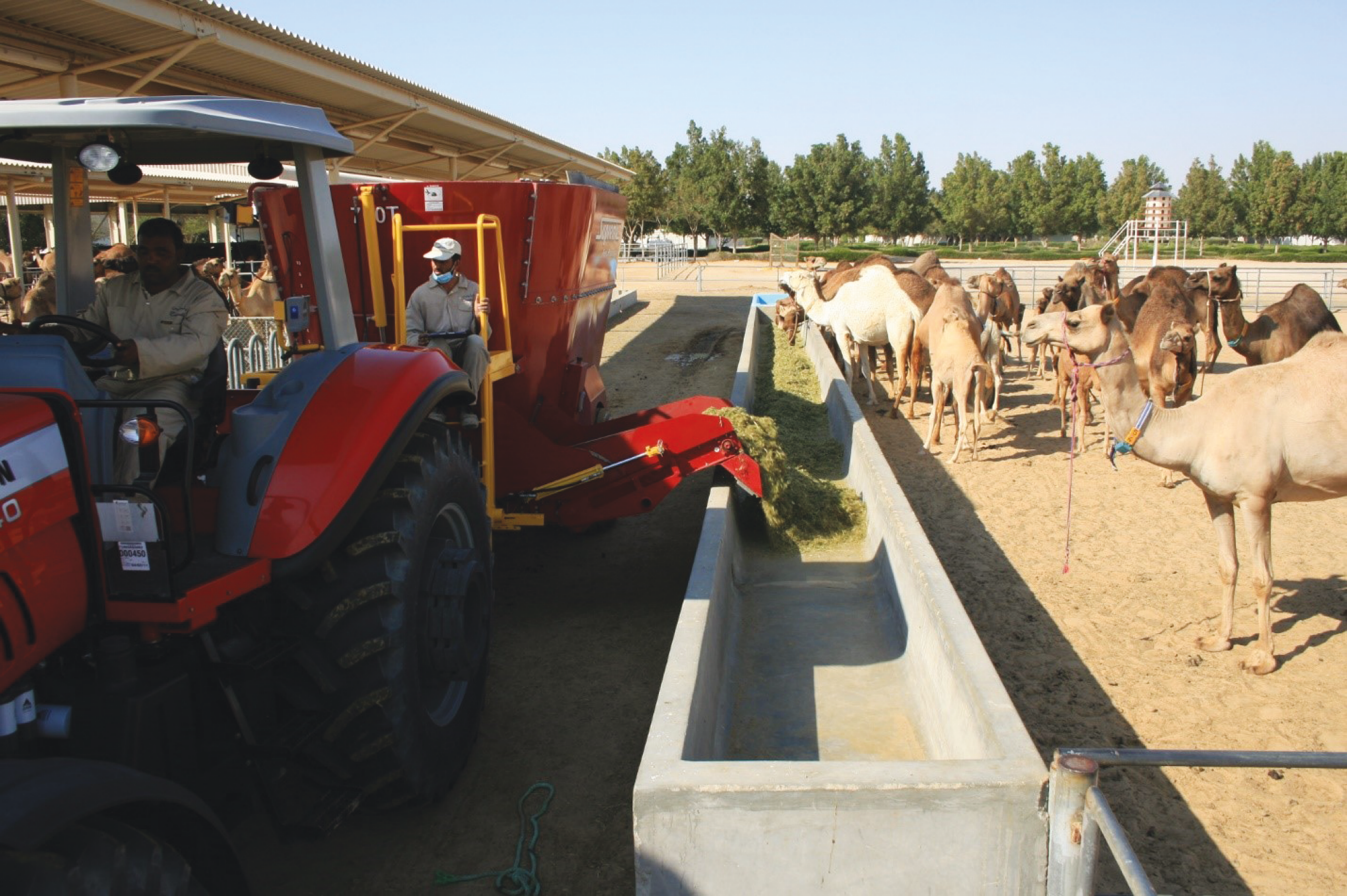
Feeding of dromedaries with a TMR (Total Mixed Ration) machine.

**Figure 5.**
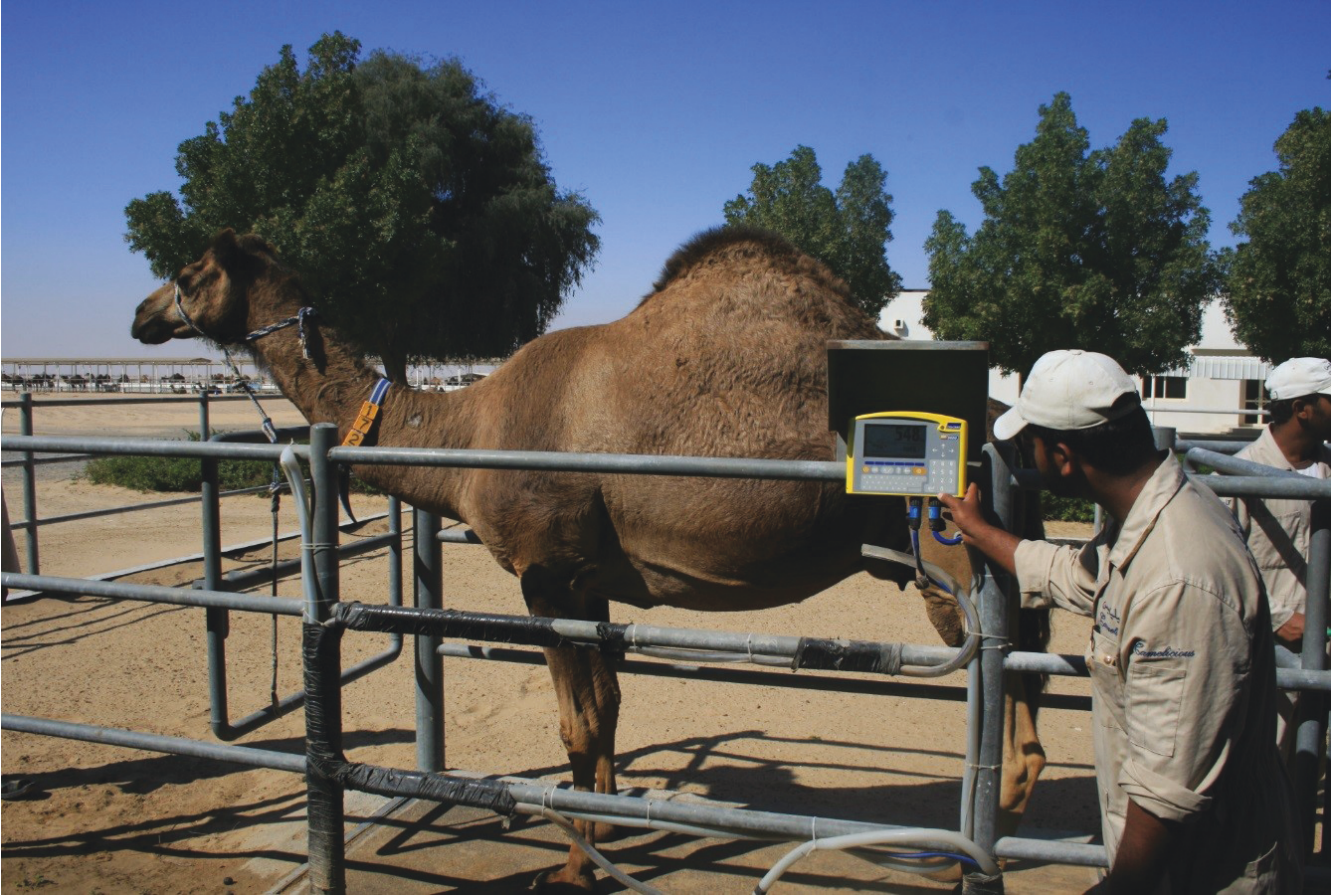
Weighing and body condition scoring of dromedaries.

As mentioned earlier in this paper, the animal welfare or well-being program (“*happy and healthy*” camels) at EICMP has been an integral part of the Herd Health Management program and it is in line with OAI recommendations (OAI, 2021). The details of “good housing” have been described earlier, but it is important to emphasize that daily cleaning of the paddocks and the environment is vital to control the transmission of diseases and fly infestation. In order to provide “good feeding”, a comprehensive feeding program taking into account the age, physiological status, production level, and genetic potential of the animals has been developed (Figure 4). The efficiency of the feeding program is evaluated by regular weighing of lactating animals and the body conditions scoring (**BCS**) of all age groups (Figure 5). This later procedure gives a great opportunity not only to verify feeding but also to have an overview on the general health status of the herd including skin condition, ectoparasites, abscesses, and nails.

The proper handling and training of animals is crucial for the entire operation and helps the camels cope with the challenges of intensive production. In hand milking, the milk let-down is triggered by the suckling effect of the calf. However, in modern milking parlors calves are not present and camels are trained to release the milk by manual and/or machine stimulation. If the training is done properly, due to their good cognitive capacity, dromedaries will remember and cooperate with all important activities for the rest of their life, such as milking, reproductive examination, and exercise. Good training is based on the fact that dromedaries are social or herd animals with extensive vocal and body language communication skills. With proper interpretation of these behavioral signs and positive reinforcement, they can be quickly and efficiently trained for different tasks. However, there are regular or occasional procedures such as samplings (blood, milk), treatments, washing for ecto-parasite control, nail trimming, and delivery assistance when individual handling and various degrees of restraint are required. This may cause temporary discomfort to the animal but it is necessary both for the safety of the camel and staff. We also have to emphasize that the intensity of vocalization is not always in proportion with the severity of the procedure or the restraint. In traditional management, the movement of animals is frequently restricted by ropes or hobbles on the front and/or hind legs (Dioli, 2022). If these ropes are too tight and left in place for too long, they cause deep wounds. However, in certain situations (like group mating of maiden females, calf rejection by delivered camels; Hamadi et al., 2021) the temporary and controlled use of hobbles can be beneficial and prevent unnecessary injuries to animals and people.

The early recognition of diseased animals both at herd and individual level is an important animal welfare issue. Camels are known to have high pain tolerance and show limited clinical signs even in serious, live threatening conditions (like intestine torsion). Therefore, the proper and continuous training of staff and the regular and frequent daily health monitoring routine are essential.

## The Role of Assisted Reproduction in the Genetic Improvement of Milk Production in Dromedaries

The genetic progress of milk production has been slow in this species due to lack of proper recording, selective breeding, and low reproductive efficiency. The latter is due to a delay in the onset of puberty (usually reaching puberty at 3–4 yr of age), a short breeding season (November–March), a long gestation period of 13 mo producing one calf per pregnancy, and a long period of lactation and calving interval. This has led to increased research into assisted reproductive techniques, such as embryo transfer and artificial insemination, to enhance reproductive efficiency, and increase the number of progeny per animal, per season. The efficiency of a genetics improvement program that includes identification and phenotypic characterization, milk data collection and analysis, reproductive data recording, and establishment of an inhouse studbook can be greatly increased by the use of assisted reproductive technologies (Nagy et al., 2013a).

### Embryo transfer

**Figure 6.**
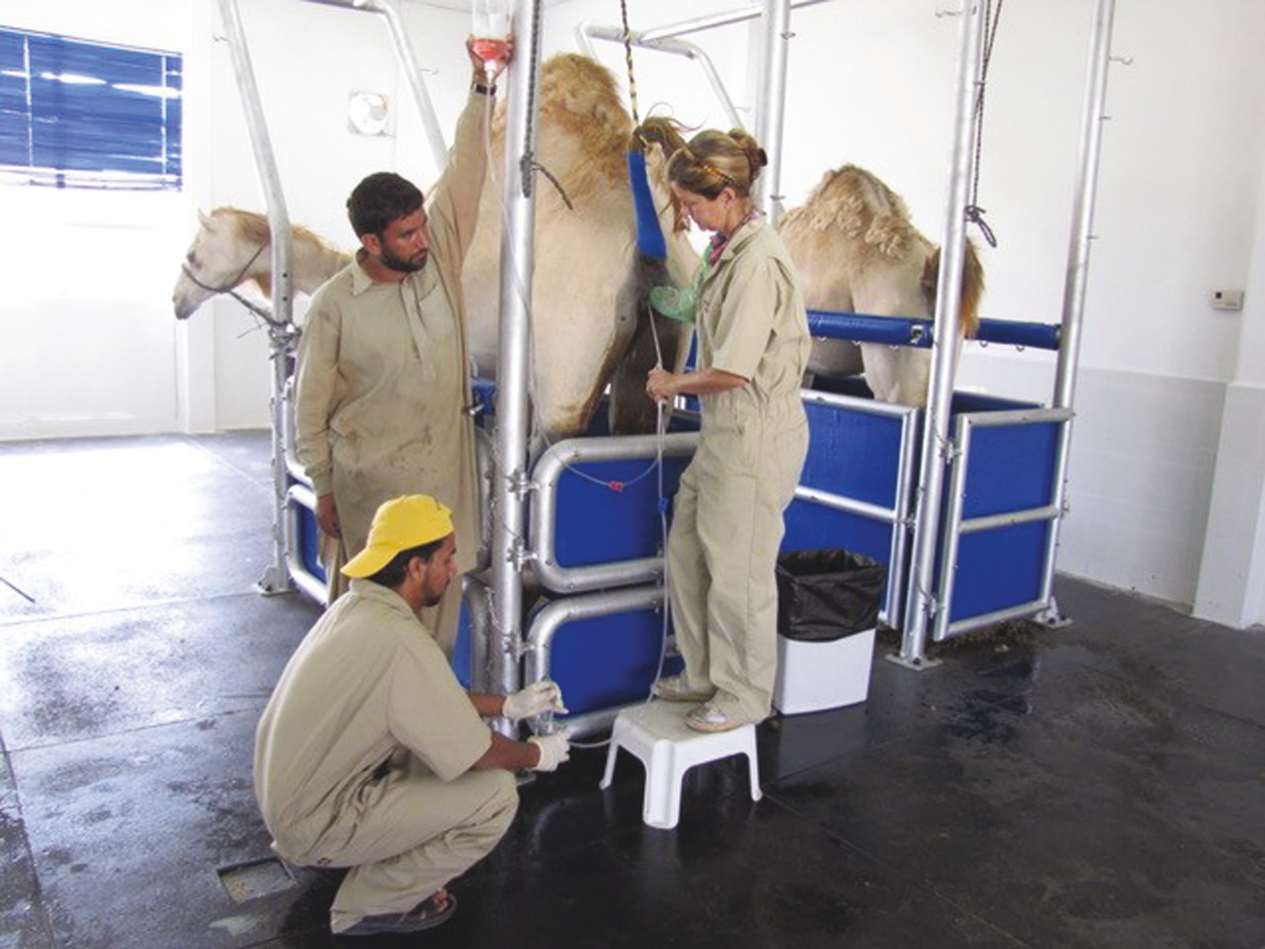
Embryo collection through transcervical flushing in an embryo transfer program.

Under natural mating conditions camels have singleton pregnancies, twins are extremely rare and, if they do go to term, they are generally born weak and under-developed. Embryo transfer therefore has several advantages as multiple progenies can be produced from desirable combinations of sire and dam in one season. Embryos can also be transported much more easily than live animals thereby diversifying the genetics worldwide, and can be stored cooled, or frozen thus preserving the genetics for many years. There are certain prerequisites for a successful embryo transfer program, namely stimulation of the donor to produce multiple follicles and synchronization of the recipients with the donor. Donors can be treated hormonally with either equine chorionic gonadotrophin (2,000–6,000 i.u. eCG;Novormon, Syntex SA, Argentina), with or without porcine FSH (400 mg pFSH; Folltropin;Vetoquinol, QC, Canada) (McKinnon et al., 1994; Skidmore et al., 2002, 2004). After 7–10 d the donor is mated and the uterus flushed, nonsurgically, 8 d after mating to recover the embryos (McKinnon et al., 1994; Skidmore et al., 2002; Figure 6). Pregnancy rates of between 65% and 75% can be obtained when these fresh embryos are transferred into recipients that have ovulated 24–48 h behind the donor. Pregnancy can then be diagnosed by ultrasonography from as early as day 18–20 of gestation (Tinson and McKinnon, 1992).

### Cryopreservation of embryos

In camel industries, there is often a considerable spatial and temporal separation of recipient and donor necessitating the need to freeze embryos so that they can be transported and transferred to recipients in a different location. Attempts to freeze camel embryos started in the late 1990’s with the first successful pregnancies reported in 2004/2005 (Skidmore et al., 2004; Nowshari et al., 2005). The principle of cryopreservation is to use cryoprotective agents (CPAs; e.g., permeating CPAs – glycerol, DMSO, and ethylene glycol, and nonpermeating CPAs – sucrose, glucose, and trehalose) to replace intracellular water from embryos and prevent the formation of ice crystals during the freezing and thawing process (Edgar et al., 2009; Edgar and Gook, 2012; Fasano et al., 2014). Initial studies used slow-freezing methods that use lower concentrations of CPA’s and slower freezing rates, but need specialized embryo freezing machines. Subsequent studies involved vitrification of embryos which is a simpler and quicker technique for cryopreservation of embryos in field conditions and, does not require the specialized equipment needed for slow cooling (Vajta, 2000; Edgar and Gook, 2012; Fasano et al., 2014). A combination of higher concentrations of CPAs and increased cooling/warming rates reduces ice crystal formation and thus improves the survival of the cells. Pregnancy rates of around 50% have been achieved with a specialized vitrification kit that was developed for camels (“Herrids Vitrification kit for camel embryos” Minitube, Germany; Skidmore et al., 2020, 2021) and although more work is required to fine-tune the protocols to increase pregnancy rates further, this kit has produced encouraging results and could lead to embryo cryopreservation being successfully incorporated into commercial embryos transfer programs for camels

## Conclusion

The milk production potential of dromedaries has not been exploited to its full capacity until recently, despite the fact that camels are an important food source in arid and semiarid regions. Camel milk has been produced exclusively by hand milking in traditional, extensive, or semiintensive farming systems, was mainly consumed locally without further processing and only a fraction of the production reached urban markets. The intensification of the camel dairy industry started 15 to 20 yr ago but such production systems are not widespread, although, it is expected to grow in the coming decades. Intensive camel milk production requires a significant initial investment, which has to include a continuous water and electricity supply, a regular and reliable feed source, as well as well-trained professionals and veterinary service, a processing facility, and reliable access to markets. Unfortunately, today these requirements are difficult to meet in most countries where camels are kept naturally. If the above conditions are available, dromedaries can be integrated efficiently into an intensive production environment. However, such a system requires the implementation and the strict execution of a Herd Health Management program and the compliance with statutory requirements in order to produce good quality and safe raw camel milk from “*happy and healthy*” animals. The experience at EICMP clearly demonstrates that intensive camel milk production is sustainable, a better than average animal health status can be maintained and the welfare requirements of the animals can also be fulfilled. In addition, no undesired effects associated with intensification such as the emergence of zoonotic diseases and antibiotic resistance were detected. Intensification also comprises the continuous improvement of milk production potential of the herd through a selective breeding program. The efficiency of this program can be substantially enhanced by the intensive use of assisted reproductive technologies.

## About the Authors

**Peter Pal Nagy**, DVM, PhD, Diplomat ECAR, hon. Professor is a veterinarian, graduated from the University of Veterinary Medicine in Budapest, Hungary in 1990. He worked in his alma mater until 1999 as assistant professor on large animal reproduction, theriogenology and endocrinology. He obtained his PhD degree in 1998 on Equine reproduction. Peter has been working with dromedary camels over 20 years. In 2000–2002, he developed an embryo transfer and artificial insemination program for dromedary camels in the Sultanate of Oman. Since 2003, he has been in Dubai, UAE to develop and manage the world first large-scale camel milking farm (EICMP, Camelicious®). Peter is a founding diplomat of the European College of Animal Reproduction (ECAR), an honorary professor at the Széchenyi István University in Hungary and chairman of the Camelid Working Group of EAAP. He is one of the founders and co-organizer of the ICAR Satellite Meetings on Camelid Reproduction.

**Dr Skidmore** graduated with a BSc (Hons) in Animal Science, (Wye College, University of London, UK. 1985), before spending five years at the Equine Fertility Unit in Cambridge, as a research associate specializing in equine reproduction. In 1991 she registered with the University of Cambridge (U.K.) and joined the Camel Reproduction Centre as a post-graduate research student, and successfully completed her PhD, “Reproduction in the dromedary camel,” in 1994 (University of Cambridge, UK). Subsequently, she has remained as Scientific Director of the Camel Reproduction Centre in Dubai, leading a team of scientists covering all aspects of camel reproductive physiology and publishing papers in ovarian follicular dynamics, pregnancy and placentation, endocrinology throughout the cycle and pregnancy, maternal recognition of pregnancy, embryo transfer and management of recipients, hybridization of New and Old World Camelids and artificial insemination and semen preservation.

**Judit Juhasz**, DVM, PhD is a veterinarian, received her degree in 1988 from the University of Veterinary Medicine in Budapest, Hungary. She worked as a reproductive specialist at the Experimental Station of the University where she developed the equine artificial insemination program. She received her PhD degree in 2002 on Equine andrology. Judit has been working with dromedary camels over 20 years. In 2000–2002, she worked in the Sultanate of Oman to develop an embryo transfer and artificial insemination program for racing camels. Since 2003, she has been in Dubai, UAE to develop, then to manage the world first large-scale camel milking farm (EICMP, Camelicious®) where she is Chief Veterinary Officer since 2006. Judit has special interest in sustainable animal welfare and large-scale farm management. She is also one of the founders and co-organizer of the ICAR Satellite Meetings on Camelid Reproduction.

## References

[CIT0001] Abdalla, E.B., A.A. Ashmawy, M.H. Farouk, O.A. Salama, F.A. Khalil, and A.F. Seioudy. 2015. Milk production potential in Maghrebi she-camels. Small Rumin. Res. 123:129–135. doi:10.1016/j.smallrumres.2014.11.004

[CIT0002] Abeiderrahmane, N . 2005. Modern dairy products from traditional camel herding: an experience in Mauritania. In: Faye, B., and P. Esenov, editors. Desertification combat and food safety, the added value of camel producers. Amsterdam, The Netherlands: NATO Science Series, IOS Press; p. 152–157.

[CIT0003] Agab, H . 2006. Diseases and causes of mortality in a camel (*Camelus dromedarius*) dairy farm in Saudi Arabia. J. Camel Pract. Res. 13(2):165–169.

[CIT0004] Ayadi, M., R.S. Aljumaah, A. Musaad, E.M. Samara, M.M. Abelrahman, M.A. Alshaikh, S.K. Saleh, and B. Faye. 2013. Relationship between udder morphology traits, alveolar and cisternal milk compartments and machine milking performances of dairy camels (*Camelus dromedarius*). Span. J. Agric. Res. 11(3):790–797. doi:10.5424/sjar/2013113-4060

[CIT0005] Boujenane, I . 2020. Review of milk let-down in camels and proposition of a milk recording method. Trop. Anim. Health Prod. 52(6):2845–2853. doi:10.1007/s11250-020-02408-1 33011907

[CIT0006] Chamekh, L., T. Khorchani, M. Dbara, M. Hammadi, and M.H. Yahyaoui. 2020. Factors affecting milk yield and composition of Tunisian camels (*Camelus dromedarius*) over complete lactation. Trop. Anim. Health Prod. 52(6):3187–3194. doi:10.1007/s11250-020-02344-0 32642909

[CIT0007] Dahiya, S.S., S. Kumar, S.C. Mehta, S.D. Narnaware, R. Singh, and F.C. Tuteja. 2016. Camelpox: a brief review on its epidemiology, current status and challenges. Acta Trop. 158:32–38. doi:10.1016/j.actatropica.2016.02.014 26902797

[CIT0008] Directive 2001/82/EC of the European Parliament and of the Council of 6 November 2001 on the Community code relating to veterinary medicinal products. 2001. Off. J. L 311:0001–0066 (ES, DA, DE, EL, EN, FR, IT, NL, PT, FI, SV).

[CIT0009] Dioli, M . 2022. Observation on dromedary (*Camelus dromedarius*) welfare and husbandry practices among nomadic pastoralists. Pastoralism. 12:7. doi:10.1186/s13570-021-00221-5

[CIT0010] Dioli, M., H.J. Schwartz, and R. Stimmelmayr 1992. Management and handling of the camel. Chapter III. In: Schwartz, H.J., and M. Dioli, editors. The one-humped camel (*Camelus dromedarius*) in Eastern Africa. A pictorial guide to disease, health care and management. Weikersheim, Germany: Margraf; p. 62–154.

[CIT0011] Edgar, D.H., and D.A. Gook. 2012. A critical appraisal of cryopreservation (slow cooling *versus* vitrification) of human oocytes and embryos. Hum. Reprod. Update. 18(5):536–554. doi:10.1093/humupd/dms016 22537859

[CIT0012] Edgar, D.H., J. Karani, and D.A. Gook. 2009. Increasing dehydration of human cleavage-stage embryos prior to slow cooling significantly increases cryosurvival. Reprod. Biomed. Online. 19:521–525. doi:10.1016/j.rbmo.2009.06.002 19909593

[CIT0013] Eleman, O.M., S.A. Omer, L.F. Albokhadaim, and A.M. Homeida. 2010. Withdrawal times of intramammary antibiotics in camel milk. Res. J. Pharmacol 4(3):83–85. doi: 10.3923/rjpharm.2010.83.85.

[CIT0014] FAO. 2020. FAOSTAT– [accessed January 25, 2022]. Available online at: https://www.fao.org/faostat/en/#data/QCL.

[CIT0015] Farm Animal Welfare Council (FAWC). 1979. Press Statement. [accessed June 14, 2022]. https://webarchive.nationalarchives.gov.uk/ukgwa/20121007104210/http://www.fawc.org.uk/pdf/fivefreedoms1979.pdf

[CIT0016] Fasano, G., N. Fontenelle, A.S. Vannin, J. Biramane, F. Devreker, Y. Englert, and A. Delbaere. 2014. A randomized controlled trial comparing two vitrification methods versus slow-freezing for cryopreservation of human cleavage stage embryos. J. Assist. Reprod. Genet. 31:241–247. doi:10.1007/s10815-013-0145-4 24317854 PMC3933602

[CIT0017] Faye, B . 2008. Dairy productivity potential of camels. In: Cardellion, R., A. Rosati, and C. Mosconi, editors. Current status of genetic resources, recording and production systems in African, Asian and American Camelids. ICAR, Rome, Italy: ICAR technical series No. 11, Proceedings of the ICAR/FAO Seminar; p. 93–104.

[CIT0018] Faye, B . 2020. How many large camelids in the world? A synthetic analysis of the world camel demographic changes. Pastoralism. 10:25. doi:10.1186/s13570-020-00176-z

[CIT0019] Faye, B., and G. Konuspayeva. 2012. The sustainability challenge of the dairy sector. The growing importance of the non-cattle milk production worldwide. Int. Dairy J. 24:50–56. doi:10.1016/j.idairyj.2011.12.011

[CIT0020] Faye, B., H. Madani, and S.A.H. El-Rouilil. 2014. Camel milk value chain in Northern Saudi Arabia. Emir. J. Food Agric. 26(4):359–365. doi:10.9755/ejfa.v26i4.17278

[CIT0021] Fischer, A., A. Liljander, H. Kaspar, C. Muriuki, H.H. Fuxelius, E. Bongcam-Rudloff, E.P. de Villiers, C.A. Huber, J. Frey, C. Daubenberger, et al. 2013. Camel Streptococcus agalactiae populations are associated with specific disease complexes and acquired the tetracycline resistance gene tetM via a Tn916-like element. Vet. Res. 44(1):86. doi:10.1186/1297-9716-44-86 24083845 PMC3850529

[CIT0022] Gil, J.D.B., P. Reidsma, K. Giller, L. Todman, A. Whitmore, and M. van Ittersum. 2019. Sustainable development goal 2: improved targets and indicators for agriculture and food security. Ambio. 48(7):685–698. doi:10.1007/s13280-018-1101-4 30267284 PMC6509081

[CIT0023] Gilbert, W., L.F. Thomas, L. Coyne, and J. Rushton. 2021. Review: mitigating the risks posed by intensification in livestock production: the examples of antimicrobial resistance and zoonoses. Animal. 15(2):100123. doi:10.1016/j.animal.2020.100123 33573940

[CIT0024] Gyuranecz, M., U. Wernery, Z. Kreizinger, J. Juhász, O. Felde, and P. Nagy. 2016. Genotyping of Brucella melitensis strains from dromedary camels (*Camelus dromedarius*) from the United Arab Emirates with multiple-locus variable-number tandem repeat analysis. Vet. Microbiol. 186:8–12. doi:10.1016/j.vetmic.2016.02.009 27016751

[CIT0025] Hammadi, M., M. Atigui, M. Ayadi, A. Barnat, A. Belgacem, G. Khaldi, and T. Khorchani. 2010. Training period and short time effects of machine milking on milk yield and milk composition in Tunisian maghrebi camels (*Camelus dromedarius*). J. Camel Pract. Res. 17(1):1–7.

[CIT0026] Hammadi, I., M. Chniter, M. Brahmi, M. Atigui, M.D. Bouzaida, M.M. Seddik, R. Nowak, G.A. María, and M. Hammadi. 2021. Mismothering and remedying the mother-young relationship in stabled dromedary camels. Appl. Anim. Behav. 243:105424. doi:10.1016/j.applanim.2021.105424

[CIT0027] Hoter, A., S. Rizk, and H.Y. Naim. 2019. Cellular and molecular adaptation of arabian camel to heat stress. Front. Genet. 19(10):588. doi:10.3389/fgene.2019.00588 PMC6593249 31275361

[CIT0028] ISO (International Organization for Standardization). 2011. ISO/TS 22002-3:2011. Prerequisite programmes on food safety - part 3: farming. Geneva, Switzerland: ISO.

[CIT0029] ISO (International Organization for Standardization). 2018. ISO 22000:2018. Food safety management systems — requirements for any organization in the food chain. Geneva, Switzerland: ISO.

[CIT0030] Iqbal, A., and B.B. Khan. 2001. Feeding behaviour of camel – review. Pak. J. Agric. Sci. 38(3-4):58–63.

[CIT0031] Jemmali, B., M.A. Ferchichi, B. Faye, and M. Kamoun. 2016. Milk yield and modeling of lactation curves of Tunisian she-camel. Emir. J. Food Agric. 28(3):208–211. doi:10.9755/ejfa.2015-07-505.

[CIT0032] Jazayeri, A., S. Shekarchian, M. Ghobadi, and M. Motaghi. 2012. Withdrawal times of intramammary antibiotics in camel and cows milk of Golestan State Iran. Res. J. Biol. Sci. 7:192–194. doi:10.3923/rjbsci.2012.192.194

[CIT0033] Juhász, J., O. Márkó, and P. Nagy. 2008. Milk production and mastitis in dromedary camels (*Camelus dromedarius*). Book of Abstracts of the 16th International Conference on Animal Reproduction. Reprod. Dom. Anim. 43(Suppl. 3):12.

[CIT0034] Juhász, J., and P. Nagy. 2012a. Development and operation of large-scale camel milking farm: challenges, experiences and results (in Hungarian). Hungarian Vet. J. 134:52–62.

[CIT0035] Juhasz, J., and P. Nagy. 2012b. Mastitis control program in a large scale camel dairy. In: Juhasz, J., J.A. Skidmore, and P. Nagy, editors. Proceedings of ICAR 2012 Satellite Meeting on Camelid Reproduction. Canada: Vancouver; p. 146–150.

[CIT0036] Juhasz, J., A.D. Gupta, R. Barua, S. Thomas, O. Márkó, and P. Nagy. 2015. The effect of different mastitis treatment protocols on antibiotic withdrawal period in lactating dromedaries (*Camelus dromedarius*). Proceedings of the 4th Conference of International Society of Camelid Research and Development. Almaty, Kazakhstan: ISOCARD; p. 196–198.

[CIT0037] Juhasz, J., S. Jose, J. Kinne, B. Johnson, S. Raja, E. Maio, R. Alkhatib, A. Premasuthan, O. Felde, M. Gyuranecz, et al. 2019. Brucella melitensis caused abortion in a serologically positive dromedary camel. J. Camel Pract. Res. 26(1):1–9. doi:10.5958/2277-8934.2019.00001.8

[CIT0038] Kagunyu, A.W., and J. Wanjohi. 2014. Camel rearing replacing cattle production among the Borana community in Isiolo County of Northern Kenya, as climate variability bites. Pastoralism. 4:13. doi:10.1186/s13570-014-0013-6

[CIT0039] McKinnon, A.O., A.H. Tinson, and G. Nation. 1994. Embryo transfer in dromedary camels. Theriogenology. 41:145–150. doi:10.1016/s0093-691x(05)80060-3

[CIT0040] Menchetti, L., B. Faye, and B. Padalino. 2021a. New animal-based measures to assess welfare in dromedary camels. Trop. Anim. Health Prod. 53(6):533. doi:10.1007/s11250-021-02978-8. 34739606 PMC8568688

[CIT0041] Menchetti, L., M. Zappaterra, L. Nanni Costa, and B. Padalino. 2021b. Application of a protocol to assess camel welfare: scoring system of collected measures, aggregated assessment indices, and criteria to classify a pen. Animals (Basel). 11(2):494. doi:10.3390/ani11020494 33668569 PMC7918070

[CIT0042] Musaad, A., B. Faye, and A.A. Nikhela. 2013. Lactation curves of dairy camels in an intensive system. Trop. Anim. Health Prod. 45(4):1039–1046. doi:10.1007/s11250-012-0331-x 23212839

[CIT0043] Nagy, P., and J. Juhász. 2016. Review of present knowledge on machine milking and intensive milk production in dromedary camels and future challenges. Trop. Anim. Health Prod. 48(5):915–926. doi:10.1007/s11250-016-1036-3 26992732

[CIT0044] Nagy, P., J.A. Skidmore, and J. Juhász. 2013a. Use of assisted reproduction for the improvement of milk production in dairy camels (*Camelus dromedarius*). Anim. Reprod. Sci. 136:205–210. doi:10.1016/j.anireprosci.2012.10.011 23146200

[CIT0045] Nagy, P., S. Thomas, O. Márkó, and J. Juhász. 2013b. Milk production, raw milk quality and fertility of dromedary camels (*Camelus dromedarius*) under intensive management. Acta Vet. Hung. 61:71–84. doi:10.1556/AVet.2012.051 23439293

[CIT0046] Nardone, A., B. Ronchi, N. Lacetera, M.S. Ranieri, and U. Bernabucci. 2010. Effects of climate changes on animal production and sustainability of livestock systems. Livest. Sci. 130 (1–3):57–69. doi:10.1016/j.livsci.2010.02.011

[CIT0047] Nawroth, C., J. Langbein, M. Coulon, V. Gabor, S. Oesterwind, J. Benz-Schwarzburg, and E. von Borell. 2019. Farm animal cognition-linking behavior, welfare and ethics. Front. Vet. Sci. 6:24. doi:10.3389/fvets.2019.00024 30838218 PMC6383588

[CIT0048] Nowshari, M.A., S.A. Ali, and S. Saleem. 2005. Offspring resulting from transfer of cryopreserved embryos in camel (*Camelus dromedarius*). Theriogenology. 63:2513–2522. doi:10.1016/j.theriogenology.2004.10.014 15910931

[CIT0049] OAI Terrestrial Animal Health Code. 2021. Volume I. Section 7. Animal Welfare. – [accessed February 24, 2022]. https://www.oie.int/en/what-we-do/standards/codes-and-manuals/terrestrial-code-online-access/.

[CIT0050] Salah, A.A., I.D. Robertson, and A.S. Mohamed. 2019. Prevalence and distribution of Trypanosoma evansi in camels in Somaliland. Trop. Anim. Health Prod. 51(8):2371–2377. doi:10.1007/s11250-019-01947-6 31177471

[CIT0051] Schmidt-Nielsen, B., K. Schmidt-Nielsen, T.R. Houpt, and S.A. Jarnum. 1956. Water balance of the camel. Am. J. Physiol. 185(1):185–194. doi:10.1152/ajplegacy.1956.185.1.185 13313770

[CIT0052] Simpkin, S.P., and P. Rowlinson. 1998. The effect of milk recording method and calf separation on determining milk yield in the camel (Camelus dromedarius). In: Bonnet, P., editor. Proceedings of the Workshop on “Dromedaries and Camels, Dairy Animals (Dromadaires et chameaux, animaux laitiers). Montpelier, France: CIRAD-EMVT Publ.; p. 111–120.

[CIT0053] Skidmore, J.A., M. Billah, and W.R. Allen. 2002. Investigation of factors affecting pregnancy rate after embryo transfer in the dromedary camel. Reprod. Fertil. Dev. 14:109–116. doi:10.1071/rd01100 12051516

[CIT0054] Skidmore, J.A., M. Billah, and N.M. Loskutoff. 2004. Developmental competence *in vitro* and *in vivo* of cryopreserved, hatched blastocysts from the dromedary camel (*Camelus dromedarius*). Reprod. Fertil. Dev. 16:605–609. doi:10.1071/rd03094 15740682

[CIT0055] Skidmore, J.A., J.L. Vaughan, and M. Herrid. 2020. Successful vitrification of dromedary camel embryos using a novel embryo vitrification kit. Anim. Reprod. Sci. 218:106483. doi:10.1016/j.anireprosci.2020.106483 32507263

[CIT0056] Skidmore, J.A., J.L. Vaughan, C.M. Malo, and M. Herrid. 2021. Comparison of two closed vitrification methods for vitrifying dromedary camel (*Camelus dromedarius*) embryos. Theriogenology. 173:123–127. doi:10.1016/j.theriogenology.2021.07.019 34371439

[CIT0057] Tinson, A.H., and A.O. McKinnon. 1992. Ultrasonography of the reproductive tract of the female dromedary camel. In: Allen, W.R., A.J. Higgins, I.G. Mayhew, D.H. Snow, and J.F. Wade, editors. Proceedings of the 1st International Camel Conference. Newmarket, UK: R&W Publications; p. 129–137.

[CIT0058] Vajta, G . 2000. Vitrification of the oocytes and embryos of domestic animals. Anim. Reprod. Sci. 60–61:357–364. doi:10.1016/s0378-4320(00)00097-x 10844207

[CIT0059] Wasfi, I.A., W.A. Al Ali, B.A. Agha, A.M. Kamel, N.A. Al Biriki, and K.M. Al Neaimi. 2012. The pharmacokinetics and metabolism of meloxicam in camels after intravenous administration. J. Vet. Pharmacol. Ther. 35(2):155–162. doi:10.1111/j.1365-2885.2011.01312.x 21635268

[CIT0060] Wernery, U . 2016. Camelid Brucellosis: a review. J. Bacteriol. Mycol. 3(1):1019. ISSN: 2471-0172.

[CIT0061] Wernery, U., J. Juhász, and P. Nagy. 2004. Milk yield performance of dromedaries with an automatic bucket milking machine. J. Camel Pract. Res. 11:51–57.

[CIT0062] Wilson, R.T . 1989. Ecophysiology of the Camelidae and desert ruminants. Berlin, New York: Springer-Verlag; p. 1–120.

